# Thoracic aortic calcification across the clinical dysglycemic continuum in a large Asian population free of cardiovascular symptoms

**DOI:** 10.1371/journal.pone.0207089

**Published:** 2019-01-04

**Authors:** Jui-Peng Tsai, Richard Kuo, Jing-Yi Sun, Chun-Ho Yun, Kuo-Tze Sung, Chuan-Chuan Liu, Jen-Yuan Kuo, Chung-Lieh Hung, Tung-Hsin Wu, Jiun-Lu Lin, Ta-Chuan Hung, Chia-Yuan Liu, Charles Jia-Yin Hou, Hung-I Yeh, Hiram G. Bezerra

**Affiliations:** 1 Department of Biomedical Imaging and Radiological Sciences, National Yang Ming University, Taipei, Taiwan; 2 Division of Cardiology, Department of Internal Medicine, Mackay Memorial Hospital, Taipei, Taiwan; 3 Department of Radiology, Mackay Memorial Hospital, Taipei, Taiwan; 4 Department of Medicine, Mackay Medical College, Taipei, Taiwan; 5 Mackay Junior College of Medicine, Nursing and Management, Taipei, Taiwan; 6 Graduate Institute of Health Care Organization Administration, College of Public Health National Taiwan University, Taipei, Taiwan; 7 Health Evaluation Center, Mackay Memorial Hospital, Taipei, Taiwan; 8 Department of Medical Technology, Yuanpei University of Science and Technology, Hsin-Chu, Taiwan; 9 Division of Cardiology, Department of Internal Medicine, Mackay Memorial Hospital, Taipei, Taiwan; 10 Division of Endocrinology and Metabolism, Department of Internal Medicine, Mackay Memorial Hospital, Taipei, Taiwan; 11 Mackay Medicine Nursing and Management College, Taipei, Taiwan; 12 Division of Gastroenterology, Department of Internal Medicine, MacKay Memorial Hospital, Taipei, Taiwan; 13 Cardiovascular Department, University Hospitals Case Medical Center, Cleveland, OH, United States of America; University of Newcastle, AUSTRALIA

## Abstract

Thoracic aortic calcification (TAC) is tightly linked to pathological atherosclerosis and associated with certain cardiovascular diseases. While diabetes mellitus (DM) is known as a coronary heart disease equivalent, we examined the presence of TAC across the dysglycemic spectrum of diabetes mellitus (DM). We consecutively studied 3003 asymptomatic ethnic Asians underwent annual cardiovacular health survey, and further categorized them into: 1) 1760 normo-glycemic, 2) 968 pre-diabetic, and 3) 274 overt DM based on dysglycemic indices and medical histories. Several TAC parameters were assessed using non-contrast multi-detector computed tomography (MDCT), and related to dysglycemic indices or diabetes mellitus status. A remarkably graded increases of adjusted total TAC calcium burden, volume and density were seen across Non-diabetes, Pre-diabetes, and diabetes mellitus categories and positively correlated with all dysglycemic profiles (all p<0.001). Multi-variate logistic and linear regression models demonstrated independent associations between greater TAC density and all dysglycemic indices (Coef: 2.5, 1.4, 6.8 for fasting, postprandial sugar and HbA1c) and diabetes mellitus status (all p<0.05). Furthermore, Receiver-operating characteristic curves (ROC) showed fasting sugar and postprandial sugar set at 103mg/dL and 111mg/dL, separately, with HbA1c set at 5.8% all predict the presence of aortic calcification. Dysglycemic status, even without overt diabetes mellitus, were tighly linked to subclinical, pathological thoracic aortic calcification.

## Introduction

Diabetes mellitus (DM) are associated with the development of atherosclerosis and increased cardiovascular mortality [1, 2]. The central key pathological role of DM involves metabolic derangements, such as metabolic syndrome (MS), central obesity, and insulin resistance [1, 2]. Vascular calcification is a later development in atherosclerosis, (e.g. coronary artery calcification [CAC] or thoracic artery calcification [TAC]) and have been used as surrogate markers for atherosclerosis [3, 4] or prognosticator for cardiovascular morbidity and mortality. [5–7] Further, the clinical use of thoracic aortic calcification (TAC) has also been reported to be an independent predictor of future CAC. [8]

Type 2 DM is well known to increase the risk of vascular calcification, especially the medial form [9, 10], though these data have never been examined well in a large asymptomatic Asian population. To date, 2 distinct morphologies of vascular calcification, either medial or intimal (atherosclerotic) location, have been well recognized [9, 10]. Further, DM is well known as coronary heart disease (CHD) equivalent, and dysglycemic status prior to overt clinical onset of DM has recently shown to cause cardiovascular events at a relatively low threshold prior to DM diagnostic criteria [11]. Based on these, the presence of dysglycemia may theoretically influence TAC during its early stage of diabetic clinical continuum. Further, the establishment of such relations may provide an alternative clinical surrogate for prediction of future coronary heart events based on the primary preventive standpoints.

In the current study, we aimed to investigate whether increased plasma glucose and HbA1c levels were independent indicators of TAC severity in a large-scale Asian population. We further explored the threshold of generating such vascular calcifications.

## Methods

### Study population

From Jan 2005 to Dec 2012, totally 3373 consecutive participants were eligible for our current work. Among them, 3111 had baseline characteristic information available for diabetic categorization or dysglycemic status evaluation (including biochemical dysglycemic indices data and clinical medical or drug histories available) and smoking dosage assessment from structured questionnaire, with 3003 had sufficient CT image quality rendered aortic calcification analysis feasible. Our current work comprised mainly Taiwanese from our single-center (MacKay Memorial Hospital). The study was approved by the Institutional Review Board of Mackay Memorial Hospital, Taipei, Taiwan (17MMHIS082e, the need for informed consent was waived by IRB) and was performed in agreement with 1990 Declaration of Helsinki and subsequent amendments. All data were analyzed retrospectively and de-identified prior to author access and analysis. The study participants underwent annual cardiovascular health survey received non-contrast multi-detector enhanced computed tomography (CT) for the assessment of cardiovascular risk by assessing coronary artery calcium scores. Study participants (n = 3003) were consecutively enrolled and further divided into three groups, using the following criteria: 1) non-dysglycemic (subjects without known type 2 diabetes mellitus with normal blood sugar, either fasting or post-prandial, and HbA1c level, n = 1760); 2) pre-diabetes; and 3) diabetes mellitus (subject with type 2 diabetes mellitus as defined by the American Diabetes Association guidelines, n = 274). [12]. Pre-diabetes was defined as having: impaired fasting glucose (IFG) [fasting plasma glucose (FPG) levels 100 mg/dl (5.6 mmol/l) to 125 mg/dl (6.9 mmol/l)], Postprandial (2-h) plasma glucose ≥140, <200 mg/dl, or HbA1c>5.7, <6.5%. Criteria for the diagnosis of diabetes mellitus includes known diabetes diagnosis by structured questionnaire or on anti-diabetes medications including: 1) HbA1c ≥6.5%. The test should be performed in a laboratory using a method that is National Glycohemoglobin Standardization Program (NGSP) certified and standardized to the Diabetes Control and Complications Trial (DCCT) assay. Or 2) FPG ≥126 mg/dl (7.0 mmol/l). Fasting is defined as no caloric intake for at least 8 h. Or 3) Postprandial (2-h) plasma glucose ≥200 mg/dl (11.1 mmol/l). The test should be performed as described by the World Health Organization, using a glucose load containing the equivalent of 75 g anhydrous glucose dissolved in water.

All subjects were free from symptoms of exercise-induced angina or any known cardiovascular disease, including peripheral artery disease, myocardial infarction, coronary arterial disease, stroke, atrial fibrillation, or prior hospitalization for congestive heart failure. Detailed physical examination was performed as well as a thorough review from structured questionnaires, which in baseline demographics and medical history including alcohol consumption, smoking and physical activity status were all obtained. All baseline characteristics and anthropometric measures, including age, body height, body weight, and waist and buttock circumference, were collected and recorded. Standardized sphygmomanometer cuff-defined resting blood pressures were measured under resting status by medical staff blinded to other test results. A 12-lead body surface electrocardiogram (ECG) was performed for all subjects.

### Biochemical data acquisition and analysis

All sample collection and analytic principles were based on the standard requirements in the Clinical Laboratory Standards Institute (CLSI) guidelines (Specimen Choice, Collection, and Handling; Approved Guideline H18-A3). To ensure accuracy, samples had repeated testing in their original tubes in a single day to avoid sample mix-up. Fasting and post-prandial glucose levels (hexokinase method), HbA1c, total cholesterol, triglyceride, uric acid (UA), blood urea nitrogen, creatinine (kinetic colorimetric assay) levels and all lipid profiles (homogeneous enzymatic colorimetric assay) were obtained using a Hitachi 7170 Automatic Analyzer (Hitachi Corp. Hitachinaka Ibaraki, Japan). High-sensitivity C-reactive protein (Hs-CRP) levels were determined by using the highly sensitive, latex particle-enhanced immunoassay Elecsys 2010 (Roche, Mannheim, Germany).

### Thoracic aortic and coronary calcification measurement

Mulit-detector computed tomography (MDCT) of the heart was performed using a 16-slice MDCT scanner (Sensation 16, Siemens Medical Solutions, Forchheim, Germany) with 16×0.75mm collimation, rotation time 420ms and tube voltage of 120kV. In one breath-hold, images were acquired from above the level of tracheal bifurcation to below the base of heart using prospective ECG triggering with the center of the acquisition at 70% of the R-R interval. Images were reconstructed from the raw data with standard kernel in 3mm thick axial, non-overlapping slices and 25cm field of view. All image analyses were performed on a dedicated workstation (Aquarius 3D Workstation, TeraRecon, San Mateo, CA, USA). Thoracic aortic (TAC) calcified lesion of the ascending or descending aorta and coronary artery calcification (CCS)in this MDCT study (S1 Fig), were defined as an area with a density >130 Hounsfield unit (HU) that covered at least 6 pixels. We used the software for the quantification of coronary calcification for TAC measurements. The Agatston calcium score was calculated by multiplying each lesion (area) by a weighted CT attenuation score in the lesion for both TAC and CCS calculation. [13] The density factor was assigned in the following manner: 1 = lesions whose maximal density was 130–199HU; 2 = lesions of 200–299HU; 3 = lesions of 300–399HU; and 4 = lesions >400HU. A total calcium score (for both Agatson and volume) was determined by summing individual lesion scores at each anatomic site. The volume and mean HU of calcium was also measured, in mm^3^ as the volumetric score. [14] In this dedicated workstation, the result page of Agatston score also demonstrated the result of “mean HU” which is the mean HU unit of all selected calcified plaques and was defined as TAC density in our current work. (S2 Fig)

### Statistical analysis

Continuous variables were expressed as mean ± standard deviation, with one-way ANOVA performed for the differences of continuous variables among 3 clinical diabetes mellitus categories (Non- diabetes, Pre- diabetes, Diabetes mellitus (Table 1), with post-hoc pair-wise comparisons made by using Bonferroni corrections. Categorical data were shown as count and percentage, with Chi-square or Fisher’s exact test. In addition, uni-variate linear regression was used to determine the relationships of the various blood sugar dysglycemic indices (HbA1c, fasting or post-prandial sugar levels) and TAC related scores. We further conducted multi-variate linear and logistic regression models to identify the associations of various blood sugar dysglycemic indices and TAC related scores with clinical co-variates as progressive control confounding factors: model 1 was adjusted for age; model 2 included the variables in model 1 and gender and BMI; model 3 included the variables in mode 1 and 2 and systolic blood pressure, history of hypertension, cardiovascular disease, smoking, LDL, HDL and eGFR. Statistical analyses were performed by IBM SPSS statistical software version 22 for Windows (IBM Corp., Armonk, New York, USA) and STATA software (version 11.0, Stata-Corp., College Station, TX, USA) and SAS (version 9.2, NC, USA). And two-tailed p<0.05 indicated statistical significance.

**Table 1:** Baseline characteristics of study subjects.

Variables N (%); Mean ± SD	Total (N = 3003)	Non-diabetes (N = 1761)	Pre-diabetes (N = 968)	Diabetes mellitus (N = 274)	p*-*value
Basic Characteristics					
Gender (male)	2259(72.5)	1240(70.4)	720(74.4)	222(81.0)*	<0.001
Age (years)	50.0±9.7	47.1±9.07*	51.6±9.27*	56.0±9.19*^†^	<0.001
Height (cm)	165.8±11.8	166.1±9.8	165.9±9.8	166.2±8.1	0.690
Weight (cm)	68.4±12.9	66.7±12.2*	70.3±12.8*	73.3±12.2*^†^	<0.001
Waist circumference (cm)	84.3±9.9	82.1±9.5*	86.5±9.4*	90.3±9.9*^†^	<0.001
BMI (kg/m^2^)	24.7±3.5	24.0±3.3*	25.4±3.6*	26.5±3.7*^†^	<0.001
**Biochemistry**					
SBP (mmHg)	123.0±16.9	119.9±16.1*	126.1±17.2*	131.2±16.6*^†^	<0.001
DBP (mmHg)	75.9±12.0	74.7±11.4*	77.6±11.7	79.0±11.3	<0.001
Heart rate (1/min)	66.9±11.3	72.7±9.5	73.9±9.9	76.0±10.7*^†^	<0.001
AC sugar (mg/dL)	101.7±22.3	93.4±7.0*	103.0±9.2*	151.1±45.5*^†^	<0.001
PC sugar (mg/dL)	123.5±48.8	108.2±26.3*	123.0±33.2*	218.1±81.0*^†^	<0.001
HbA1C (%)	5.86±0.87	5.4±0.3*	6.0±0.4*	7.5±1.6*^†^	<0.001
Total cholesterol (mg/dL)	202.0±36.6	199.2±34.7	208.1±38.6*	198.0±38.0^†^	<0.001
Triglyceride (mg/dL)	141.7±116.7	124.9±76.3*	158.8±153.5*	189.8±154.1*^†^	<0.001
LDL cholesterol (mg/dL)	130.8±32.3	128.9±31.6*	136.0±32.2*	124.0±33.8*	<0.001
HDL cholesterol (mg/dL)	52.3±13.9	54.2±14.4*	50.3±12.5*	47.2±12.9*^†^	<0.001
Uric acid (mg/dL)	6.0±1.4	5.9±1.5	6.2±1.4*	6.0±1.4	<0.001
sGPT (U/L)	31.0±26.5	28.9±25.5*	33.2±27.5*	37.5±27.3*	<0.001
eGFR (mL/min/1.73m^2^)	82.5±15.1	83.4±14.6*	81.1±14.7	81.2±18.9	<0.001
**Health history**					
Hypertension (%)	514(17.1%)	188(10.7%)	200(20.7)	126(46.0)	<0.001
Hyperlipidemia (%)	217(5.3%)	52(3.0)	59(6.0)	49(17.9)	<0.001
CVD (%)	135(4.5%)	50(2.8)	45(4.7)	40(14.6)	<0.001
Active Smoking (%)	363(12.1%)	204(11.6)	114(11.8)	45(16.4)	0.069

*Significantly differences with non-diabetes, p<0.05

^†^ Significantly differences with pre-diabetes, p<0.05

Abbreviation: N, number; M, mean; SD, standard deviation; BMI, body mass index; DBP, diastolic blood pressure; sGPT serum glutamate pyruvate transaminase; eGFR, estimated glomerular filtration rate; HbA1C, glycosylated hemoglobin level; HDL, high-density lipoprotein; LDL, low-density lipoprotein; SBP, systolic blood pressure; PCF, Peri-cardial fat; TAT, Thoracic peri-aortic adipose tissue.

## Results

### Basic characteristics of study subjects

Among totally 3003 final study subjects with thoracic aortic calcification data analyzable, we observed a male predominance (2182, 72.7%), with a mean age of 49.7±5.7years. Of all, 1761 (58.6%) were non-diabetic, 968 (32.2%) were pre-diabetic, and 274 (9.1%) were diabetes mellitus (Table 1), by using both history and all dysglycemic lab criteria. Diabetes mellitus were significantly different from non- and pre-diabetics and tended to be older, having greater body weight/body size (BMI), greater waist circumference, higher blood pressure and tended to have higher resting heart rate (all p<0.05). Further, diabetic subjects also had significantly higher sugar levels, higher sGPT, higher HbA1c, and presented with markedly higher triglyceride though lower HDL level (all p<0.05). A significantly higher proportion of hypertension, hyperlipidemia, and coronary artery disease (CAD) were also observed in diabetes mellitus group, which was accompanied by higher smoking rate and more frequent use of alcohol (all p<0.05).

### The associations among various dysglycemic indices, diabetes mellitus and TAC

In uni-variate linear analysis (Table 2), both fasting and postprandial blood sugar levels, clinical diabetes mellitus diagnosis (crude OR: 4.98, [95% CI: 3.40–7.28], S1 Table) and HbA1c levels were all significantly associated with higher TAC calcium scores, volumes, TAC density, and the presence of aortic calcification (all p<0.05). In multi-variate regression models (Table 2), postprandial glucose level and diabetes mellitus diagnosis remained significantly associated with higher TAC calcium scores and volumes after accounting for age, though these relationships became attenuated in the final fully adjusted models. Further, all dysglycemic indices and clinical diabetes mellitus were significantly associated with higher TAC density (Coef: 2.52, 1.36, 6.82, and 14.88), and existence of thoracic aortic calcification (OR: 1.06, 1.02, 1.21, and 1.66 for fasting, postprandial sugar, HbA1c and diabetes mellitus status, respectively, all p<0.05).

**Table 2:** Linear regression analysis associated with blood sugar and TAC related score (N = 3003).

	TAC Score	TAC Volume	TAC Density	Presence of Calcification
	Coefficients (95% CI)	Coefficients (95% CI)	Coefficients (95% CI)	Odds (95% CI)
**Uni-variate model**				
AC Sugar (+10mg/dl)	18.12 (8.18, 28.06)	15.32 (7.41, 23.24)	7.04 (5.44, 8.64)	1.16 (1.12, 1.21)
PC Sugar (+10mg/dl)	16.21 (9.91, 22.52)	13.32 (8.28, 18.36)	4.88 (3.97, 5.79)	1.10 (1.08, 1.13)
HbA1C (+1%)	71.07 (32.85, 109.29)	59.55 (29.27, 89.83)	25.05 (19.65, 30.44)	1.68 (1.48, 1.91)
Diabetes **mellitus**	278.2 (153.3, 403.2)	234.6 (143.6, 325.5)	89.4 (70.7, 108.2)	3.62 (2.77, 4.72)
**Multi-variate models**				
**Model 1**^**a**^				
AC Sugar (+10mg/dl)	6.31 (-3.64, 16.25)	5.54 (-2.37, 13.44)	2.78 (1.33, 4.23)	1.08 (1.04, 1.13)
PC Sugar (+10mg/dl)	6.83 (0.29, 13.37)	5.68 (0.46, 10.91)	1.65 (0.79, 2.50)	1.04 (1.01, 1.06)
HbA1C (+1%)	23.99 (-15.01, 63.01)	20.52 (-10.33, 51.37)	8.81 (3.87, 13.74)	1.29 (1.13, 1.48)
Diabetes **mellitus**	147.1 (22.6, 271.7)	129.5 (39.3, 219.7)	43.6 (26.7, 60.4)	1.86 (1.36, 2.54)
**Model 2**^**b**^				
AC Sugar (+10mg/dl)	7.87 (-2.43, 18.17)	6.92 (-1.25, 15.1)	3.10 (1.61, 4.59)	1.08 (1.04, 1.13)
PC Sugar (+10mg/dl)	7.24 (0.63, 13.85)	6.04 (0.76, 11.31)	1.65 (0.78, 2.51)	1.03 (1.007, 1.06)
HbA1C (+1%)	24.79 (-15.43, 65.02)	21.09 (-10.70, 52.88)	8.63 (3.56, 13.69)	1.26 (1.10, 1.45)
Diabetes **mellitus**	106.7 (-21.21, 225.5)	86.4 (-8.20, 181.0)	40.9 (23.6, 58.2)	1.78 (1.28, 2.46)
**Model 3**^**c**^				
AC Sugar (+10mg/dl)	5.88 (-5.28, 17.04)	5.45 (-3.41, 14.31)	2.52 (0.95, 4.08)	1.06 (1.02, 1.11)
PC Sugar (+10mg/dl)	5.20 (-1.70, 12.1)	4.45 (-1.06, 9.96)	1.36 (0.49, 2.23)	1.02 (1.00, 1.05)
HbA1C (+1%)	12.14 (-28.75, 53.04)	11.13 (-21.18, 43.44)	6.82 (1.73, 11.90)	1.21 (1.04, 1.40)
Diabetes **mellitus**	23.33 (-82.32, 128.9)	19.26 (-64.62, 103.15)	14.88 (0.1, 29.67)	1.66 (1.16, 2.38)

^a^ Adjusted for age.

^b^ Adjusted for age, gender and BMI

^c^ Adjusted for age, gender, BMI, systolic blood pressure, history of hypertension, cardiovascular disease, smoking, LDL, HDL and eGFR.

### The thresholds and cutoff of various dysglycemic indices and TAC

The single use of fasting or post-prandial blood sugar and HbA1c in identifying the existence of thoracic aortic calcification in our current work yielded a c-statistics of 0.64 (95% CI: 0.61–0.67), 0.68 (95% CI: 0.65–0.71) and 0.68 (95% CI: 0.65–0.72) (S1 Table). Fasting sugar set at 103mg/dL (sensitivity: 47.5%, specificity: 75.4%), postprandial sugar set at 111mg/dL (sensitivity: 70.3%, specificity: 56.9%), and HbA1c set at 5.8% (sensitivity: 65.5%, specificity: 61.9%) were shown to be the most optimal cut-off with in identifying aortic calcification. Comparisons of c-statistics for detecting coronary calcification (CCS) and TAC using various dysglycemic indices were further displayed in S3 Fig. In general, a higher c-statistics were observed for identifying TAC using different dysglycemic indices compared to CCS.

### The associations among stratified various dysglycemic indices, diabetes mellitus and TAC

**Fig 1:**
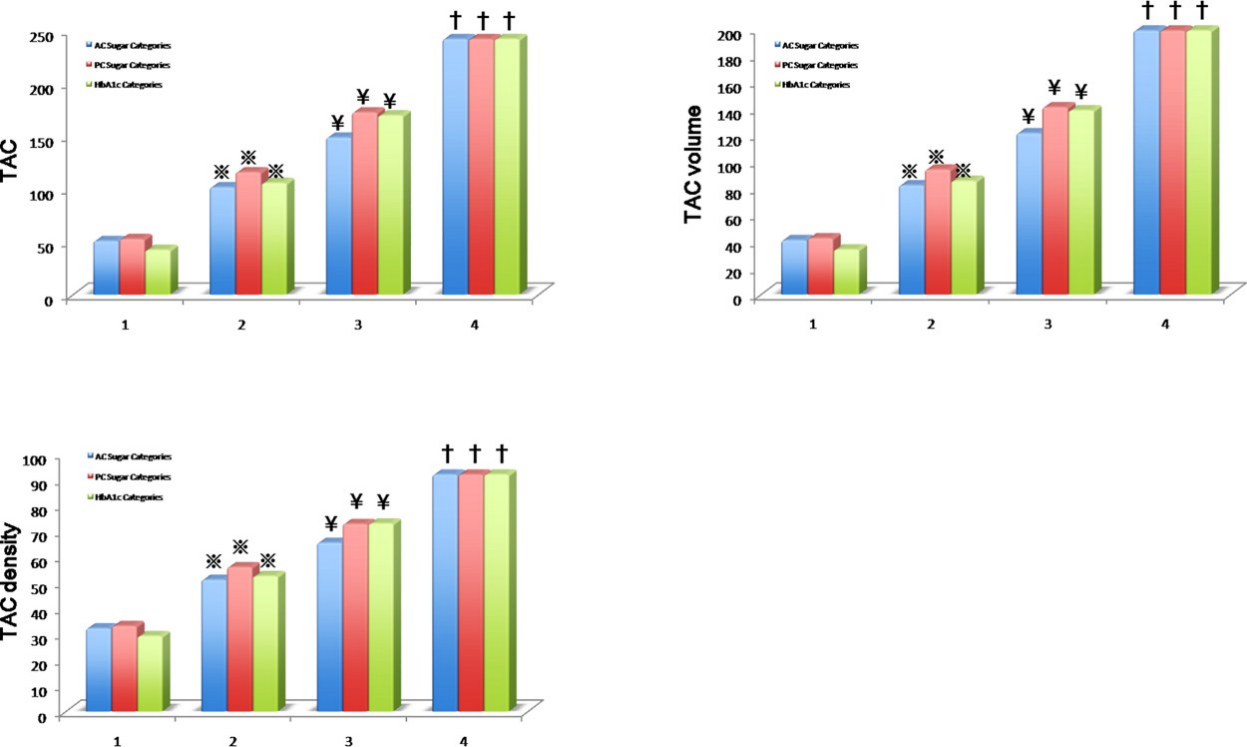
TAC, total plaque burden and mean density of plaques across dysglycemic categories. The adjusted mean values of Agaston score (TAC), total plaque volume and mean density of plaques across fasting (AC), post-prandial sugar (PC) and HbA1c categories for normo-glycemic (Group 1), pre-diabetes (Group 2), diabetes (Group 3) based on current study defined criteria of both biochemical cut-offs and history. Additionally, we further categorized those study participants with known diabetes mellitus history or known medication usage as Group 4, regardless of biochemical information. Group 4 may indicate a specific group with longstanding diabetes or diabetic subjects with chronicity. 1: Group 1, 2: Group 2, 3: Group 3, and 4: Group 4.

By stratifying the study participants based on glycemic control cut-off for both fasting and postprandial glucose levels, HbA1c, and known past diabetes mellitus history or medication use (without adding blood sampling criteria), we observed significant increase of all TAC indices relevant to higher fasting (using <100mg/dL group as reference) or postprandial glucose (using <140mg/dL group as reference), as well as higher HbA1c categories (using HbA1c<5.7% group as reference) in unadjusted models (all p<0.05, Table 3). These graded changes remained relatively unchanged after accounting for baseline clinical co-variates (except for higher HbA1c categories vs TAC score and volume) (Fig 1). The linear relationships among various dysglycemis indices and different TAC scores were further illustrated in S4 Fig (all p<0.05). Further, consistent and graded increases of adjusted means in these TAC measures (including TAC score, volume and density) were also observed (S1–S4 Tables, all trend p: <0.001).

**Table 3:** Diabetes Hx/Med: Known past diabetes history or medication use during study subjects recruitment.

**AC Sugar Stratification, mg/dl**					**AC Sugar Stratification, mg/dl**				
**Unadjusted**	**< 100**	**100 ~ 126**	**≥ 126**	**Diabetes Hx/Med**	**Adjusted**	**< 100**	**100 ~ 126**	**≥ 126**	**Diabetes Hx/Med**
**(number)**	1,800	882	140	122	**(number)**	1,800	882	140	122
**TAC score**	Reference	40.6(-9.4~90.3)	201.9(96.1~307.7)	339.7(226.4~453.0)	**TAC score**	Reference	-1.0(-53.6~55.7)	167.7(46.4~288.9)	234.5(20.4~448.6)
**TAC volume**	Reference	33.9(-5.9~73.7)	169.0(84.6~253.4)	283.1(192.8~373.5)	**TAC volume**	Reference	0.6(-42.8~44.0)	140.8(44.5~237.0)	187.4(17.4~357.3)
**TAC density**	Reference	23.6(15.7~31.5)	54.4(37.6~71.1)	102.7(84.8~120.6)	**TAC density**	Reference	6.6(-1.0~14.2)	32.9(16.0~49.8)	60.9(31~90.8)
**PC Sugar Stratification, mg/dl**					**PC Sugar Stratification, mg/dl**				
**Unadjusted**	**< 140**	**140 ~ 200**	**≥ 200**	**Diabetes Hx/Med**	**Adjusted**	**< 140**	**140 ~ 200**	**≥ 200**	**Diabetes Hx.Med**
**(number)**	1,620	620	129	122	**(number)**	1,620	620	129	122
**TAC score**	Reference	65.5(5.4~125.5)	242.8(127.3~358.3)	342.6(223.7~461.6)	**TAC score**	Reference	14.9(-48.7~78.5)	189.0(59.7~318.2)	247.0(24.5~469.6)
**TAC volume**	Reference	52.8(5.07~100.5)	204.8(113.0~296.5)	285.6(191.1~380.2)	**TAC volume**	Reference	11.4(-38.9~61.7)	160.3(58.1~262.4)	198.4(22.5~374.4)
**TAC density**	Reference	22.4(13.1~31.8)	61.4(43.4~79.3)	99.1(80.6~117.6)	**TAC density**	Reference	0.34(26.0~87.1)	30.1(12.4~47.8)	56.5(26.0~87.1)
**HbA1c Stratification**					**HbA1c Stratification**				
**Unadjusted**	**< 5.7%**	**5.7 ~ 6.5%**	**≥ 6.5%**	**Diabetes Hx/Med**	**Adjusted**	**< 100**	**100 ~ 126**	**≥ 126**	**Diabetes Hx/Med**
**(number)**	1,718	921	185	122	**(number)**	1,718	921	185	122
**TAC score**	Reference	67.1(17.5~116.7)	142.2(48.3~236.1)	347.6(234.0~461.2)	**TAC score**	Reference	11.2(-42.7~65.1)	54.1(-53.5~161.7)	178.2(-36.1~392.5)
**TAC volume**	Reference	57.7(18.2~97.3)	112.8(37.9~187.7)	289.8(199.2~380.4)	**TAC volume**	Reference	12.2(-30.5~55.0)	38.3(-47.1~123.7)	135.9(-34.2~306.1)
**TAC density**	Reference	24.5(16.7~32.4)	63.2(48.4~78.0)	104.6(86.7~122.5)	**TAC density**	Reference	2.71(-4.8~10.2)	32.2(17.2~47.2)	61.7(31.9~91.6)

## Discussion

In this large, asymptomatic adult population, we demonstrated that plasma glucose and HbA1c were both independent indicators of the development of TAC, even at levels lower than currently established diabetes mellitus threshold. Higher sugar levels, HbA1c, and presence of diabetes mellitus history all contributed to certain higher thoracic arterial calcification index, which remained unchanged even after adjustment for clinical co-variates and risk factors. Further, we also demonstrated that such arterial calcification process may start at a relatively low sugar and HbA1c level, even within pre-diabetic stage. To our best knowledge, our study is the first to establish such links in a large asymptomatic Asian population without active cardiovascular diseases and therefore is primary preventive.

TAC has been shown to be an indicator of CAC [5, 8], a marker for generalized atherosclerosis process. [15] CAC, in turn, predicts future coronary heart disease. [6, 16, 17] The sequence from normal coronary arteries to intimal atherosclerosis and calcification accumulation can be described as the following order. [18] At portions of arterial tree subjected to turbulent blood flow, endothelial cells become impaired in their ability to synthesize nitrous oxide, and further triggers activated inflammatory response with migration of monocytes and macrophages. Plaque formation follows–intimal thickening of the blood vessel walls, together with accumulation of lipoproteins by recruited macrophages/monocytes and vascular smooth muscle cells (VSMC), leading to a protective fibrous cap. [18] Finally, VSMC accumulation of lipoproteins leads to apoptosis which produces plaque calcification. [18, 19] On the other hand, it has been proposed that human thoracic or abdominal aorta is composed of different layers, including intima and media, with aging, smoking and diabetes mellitus primarily affects media layer (tunica media) calcification. [10, 20–22] Therefore, the pathological process of vascular calcification in thoracic aorta varies from intimal conditions seen in coronary arteries, and shoould be regarded as a distinct type of pathological calcification process. [23]

DM, MS, and insulin resistance (IR) have all been shown to contribute to atherosclerosis [1, 2, 24] and coronary plaque calcification. [9, 25–27] DM is thought to do this by increasing the recruitment of monocytes and macrophages to plaque sites and hence up-regulating the number of adhesion molecules on the endothelial cells. This increases the inflammation that drives the atherosclerotic process, namely intimal lesions, which primarily seen in coronary or carotid sites with progressive vascular calcification. [28] On the meanwhile, DM and HbA1c level, an averaged marker reflecting more chronic dysglycemic status, have also been shown to contribute to aortic medial layer calcification as a distinct alternative pathological process by medial layer pathology. [10, 29–31] Medial layer calcification has been proposed to be a hydroxyapatite mineralization process within the tunica media layer, [29] in part due to loss of inhibitors of mineralization such as MGP (matrix Gla protein) and pyrophosphate. [32, 33] Necrotic vascular mesenchymal cells provide substrate of phospholipid-rich debris to nucleate apatite formation. [34]

In addition, osteogenic mechanisms from altered bone-forming proteomic expressions may also play a role in vascular medial calcification. [15] Excessive accumulation of free radicals in hyperglycemia status, for example, superoxide anion, rendering activation of cellular, pathways involving polyol (which may convert glucose to sorbitol utilizing nicotinamide adenine dinucleotide phosphate hydrogen [NADPH] causing decreased antioxidant glutathione concentration and reserve) and hexosamine flux and advanced glycation end products (AGEs), protein kinase C (PKC) and possibly up-regulated nuclear factor-κB mediated vascular inflammation. [35] Additionally, hyperglycemia further increases glucose oxidation in the citric acid cycle from over-production of mitochondrial reactive oxygen species. [35] Finally, endothelial dysfunction in diabetic status may promote endothelial permeability, and thus exposing VSMCs directly to hyperglycemic plasma and some other pro-inflammatory circulating factors, together with over-expression of tumor necrosis factor-α, both further enhance medial calcification process. [36]

**Fig 2:**
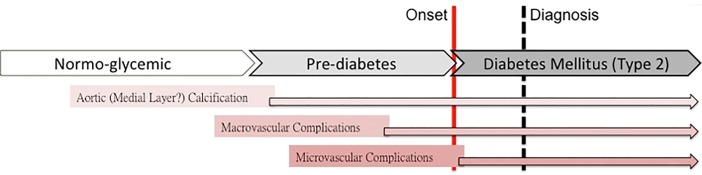
Hypothetical scheme of the pathophysiology of dysglycemic continuum. Hypothetical scheme for the pathophysiology of dysglycemic continuum of micro-, macrovacular complications and findings of medial layer calcification of thoracic aorta. Arterial calcification may serve as an alternative, clinical early marker of vascular complications in dysglycemic subjects based on current study.

Except for well-known macrovascular complications myocardial infarction or stroke associated with diabetes mellitus, calcification of the aorta is also known as a marker for atherosclerosis [37–39], which probably partly mediates aortic dissection pathologies. [40] Therefore, as mentioned above, aortic calcification can be clinically used as an alternative surrogate for incident cardiovascular disorders likely to happen in an earlier and pre-clinical stage of diabetes mellitus (Fig 2). In a recent large Chinese cohort, random sugar sampling set at 106mg/dL had been shown to be associated 11% higher risk for cardiovascular death. In a diabetes and heart study, Churchill et al reported that calcifications of ascending and descending thoracic aorta were independently associated with Caucasian race and diabetes duration [41]. In our work, we have shown that either increased plasma glucose itself or HbA1c level may serve as independent factor associated with great artery calcifications [11] in terms of increased TAC at a level within pre-DM range in a large Chinese population free of cardiovascular symptoms, which further supplemented and expanded prior literatures into a broader population. In this regard, our data indicated that vascular medial calcification may start at a relative early stage process of dysglycemic spectrum. These results may suggest the need for more aggressive actions of preventive therapy for subjects with more early stage dysglycemia with higher risks.

There are several limitations in our study. Firstly, all study participants were enrolled consecutively from cardiovascular health survey in a single center. Secondly, our cohort included more male subjects than females which may limit its generalizability. Besides, this is a retrospective, cross-sectional study; further longitudinal studies are necessary to confirm the relationships among higher TAC, greater cardiometabolic risks and relevant prognostic value. Finally, information about the clinical onset and duration of diabetes mellitus was not available in our current work, which preclude the feasibility on analysis of dysglycemic chronicity on aortic calcification burden.

## Supporting information

S1 Fig Thoracic aortic calcifications by 2D and 3D computed tomography views. MDCT demonstrated thoracic aorta with and without calcified plaques. 44 y/o male with normo-glycemia and no aortic calcification in axial view (A), sagittal view (B) and 3d reconstruction image (C); 70 y/o male with hyperglycemia and aortic calcification with arrow pointed in (D), (E) and (F).
(TIF)

S2 Fig Illustrations of thoracic aortic calcification analysis. Aortic calcification analysis by the software showed “pink” color labelled aortic calcified plaques (arrowheads, right-sided CT axial image) and results of Agaston score (TAC), total plaque volume and mean density of plaques (left-sided column).
(TIF)

S3 Fig c-statistics of various dysglycemic indices and adjusted odds ratio (OR) for presence of thoracic aortic or coronary calcification. Comparisons of c-statistics for various dysglycemic indices(A), and adjusted odds ratio (OR) for presence of diabetes mellitus (defined by combined dysglycemic indices, history and medication use, or simply history and medication use) (B) in identifying thoracic aortic or coronary calcification.
(TIF)

S4 Fig Linear relationships between various higher dysglycemic indices and TAC scores. (TIF)

S1 Table Receiver operating characteristic curves and c-statistics of various dysglycemic indices on the presence of thoracic aortic calcification. (DOCX)

S2 Table Comparison of differences between different levels of AC sugar and TAC related score. (DOCX)

S3 Table Comparison of differences between different levels of PC sugar and TAC related score. (DOCX)

S4 Table Comparison of differences between different levels of HbA1C and TAC related score. (DOCX)
