# Correction: Thoracic aortic calcification across the clinical dysglycemic continuum in a large Asian population free of cardiovascular symptoms

**DOI:** 10.1371/journal.pone.0214186

**Published:** 2019-03-18

**Authors:** Jui-Peng Tsai, Richard Kuo, Jing-Yi Sun, Chun-Ho Yun, Kuo-Tzu Sung, Chuan-Chuan Liu, Jen-Yuan Kuo, Chung-Lieh Hung, Tung-Hsin Wu, Jiun-Lu Lin, Ta-Chuan Hung, Chia-Yuan Liu, Charles Jia-Yin Hou, Hung-I Yeh, Hiram G. Bezerra

The fifth author’s name is spelled incorrectly. The correct name is: Kuo-Tzu Sung.

Two affiliations for the first author are missing. Jui-Peng Tsai is affiliated with 1, 2, 4, and 5. #1 Department of Biomedical Imaging and Radiological Sciences, National Yang Ming University, Taipei, Taiwan. #2 Division of Cardiology, Department of Internal Medicine, Mackay Memorial Hospital, Taipei, Taiwan. #4 Department of Medicine, Mackay Medical College, Taipei, Taiwan. #5 Mackay Junior College of Medicine, Nursing and Management, Taipei, Taiwan.

The number of normo-glycemic survey results in the third sentence of the Abstract is incorrect. There we 1761 normo-glycemic results. The correct sentence is: “We consecutively studied 3003 asymptomatic ethnic Asians underwent annual cardiovacular health survey, and further categorized them into: 1) 1761 normo-glycemic, 2) 968 pre-diabetic, and 3) 274 overt DM based on dysglycemic indices and medical histories.”

Coronary artery calcification (CAC) appears incorrectly as coronary calcification (CCS) throughout the manuscript. Accordingly, the following sentences must be corrected.

The fifth, sixth, and seventh sentences of the “thoracic aortic and coronary calcification measurement” section of the Methods are incorrect. The correct sentences are: “Thoracic aortic (TAC) calcified lesion of the ascending or descending aorta and coronary artery calcification (CAC) in this MDCT study (S1 Fig), were defined as an area with a density >130 Hounsfield unit (HU) that covered at least 6 pixels. We used the software for the quantification of coronary artery calcification for TAC measurements. The Agatston calcium score was calculated by multiplying each lesion (area) by a weighted CT attenuation score in the lesion for both TAC and CAC calculation.”

The last two sentences of the “the thresholds and cutoff of various dysglycemic indices and TAC” section of the Results are incorrect. The correct sentences are: “Comparisons of c-statistics for detecting coronary artery calcification (CAC) and TAC using various dysglycemic indices were further displayed in S3 Fig. In general, a higher c-statistics were observed for identifying TAC using different dysglycemic indices compared to CAC.”

There is a typographical error in the fourth sentence of the second paragraph of the Discussion section. The correct sentence is: “Plaque formation follows intimal thickening of the blood vessel walls, together with accumulation of lipoproteins by recruited macrophages/monocytes and vascular smooth muscle cells (VSMC), leading to a protective fibrous cap.”

There is a typographical error in the final sentence of the second paragraph of the Discussion section. The correct sentence is: “Therefore, the pathological process of vascular calcification in thoracic aorta varies from intimal conditions seen in coronary arteries, and should be regarded as a distinct type of pathological calcification process.”
