# Supplementary figures and images for: Thoracic aortic calcification across the clinical dysglycemic continuum in a large Asian population free of cardiovascular symptoms
Source: PLoS One. 2019 Jan 4;14(1):e0207089. doi: 10.1371/journal.pone.0207089 (PMC6319708; doi:10.1371/journal.pone.0207089)

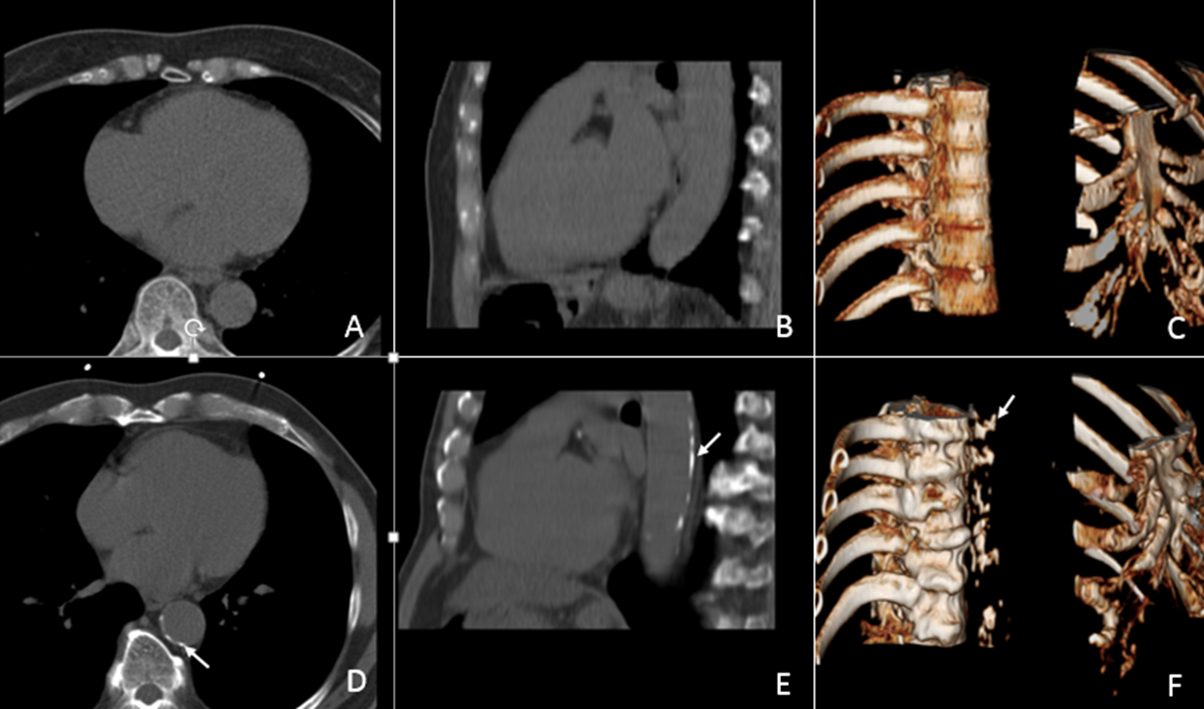

Supplement: S1 Fig — MDCT demonstrated thoracic aorta with and without calcified plaques. 44 y/o male with normo-glycemia and no aortic calcification in axial view (A), sagittal view (B) and 3d reconstruction image (C); 70 y/o male with hyperglycemia and aortic calcification with arrow pointed in (D), (E) and (F). (TIF) [file pone.0207089.s001.tif]

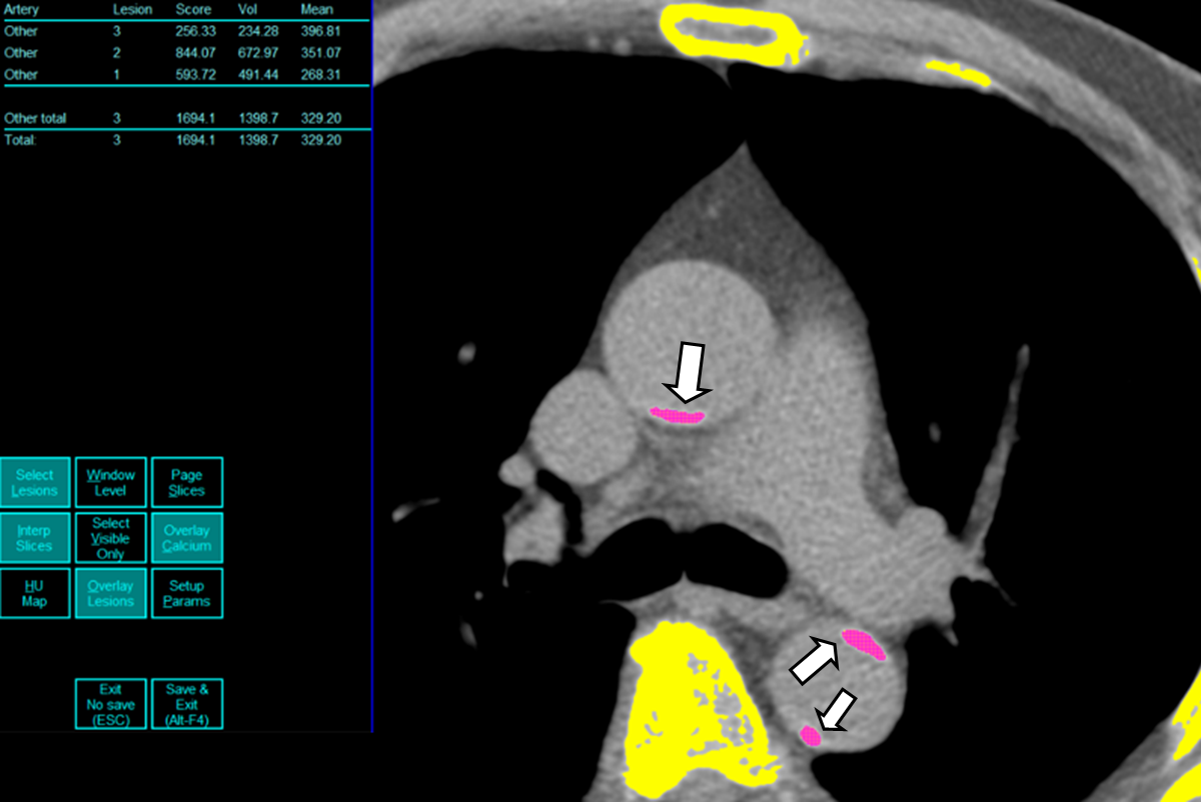

Supplement: S2 Fig — Aortic calcification analysis by the software showed “pink” color labelled aortic calcified plaques (arrowheads, right-sided CT axial image) and results of Agaston score (TAC), total plaque volume and mean density of plaques (left-sided column). (TIF) [file pone.0207089.s002.tif]

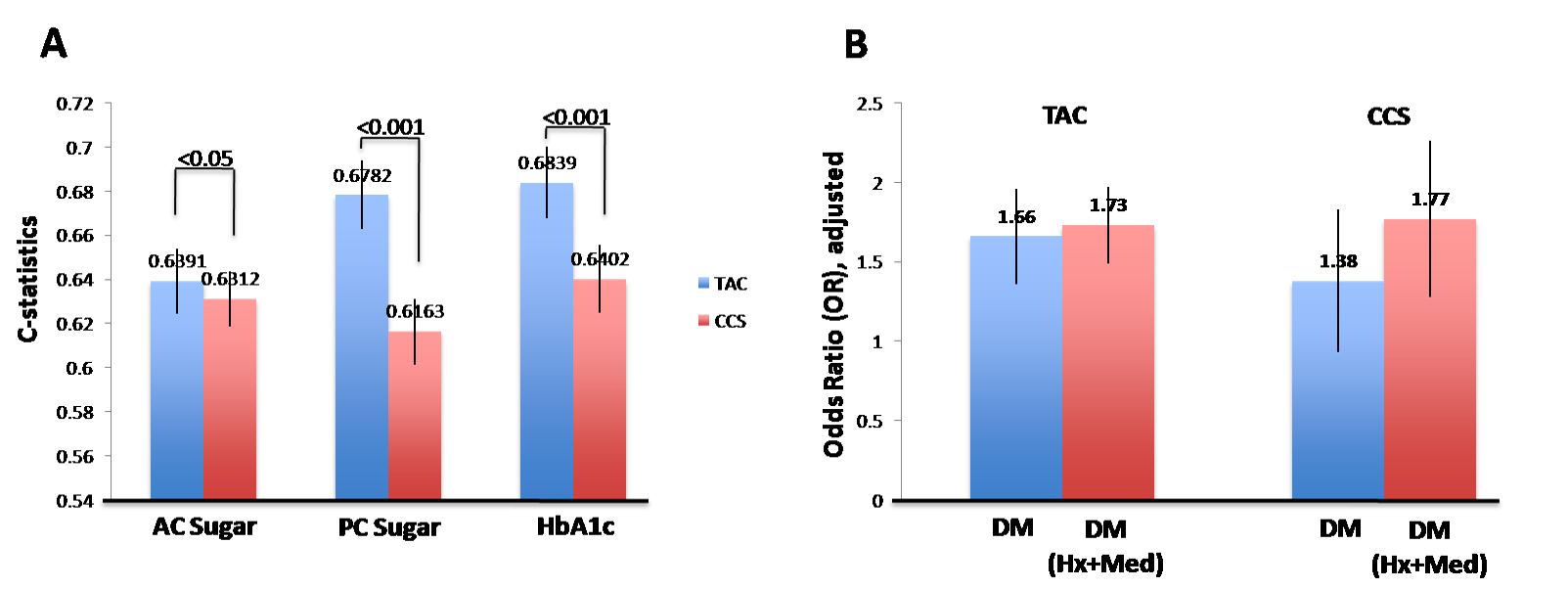

Supplement: S3 Fig — Comparisons of c-statistics for various dysglycemic indices(A), and adjusted odds ratio (OR) for presence of diabetes mellitus (defined by combined dysglycemic indices, history and medication use, or simply history and medication use) (B) in identifying thoracic aortic or coronary calcification. (TIF) [file pone.0207089.s003.tif]

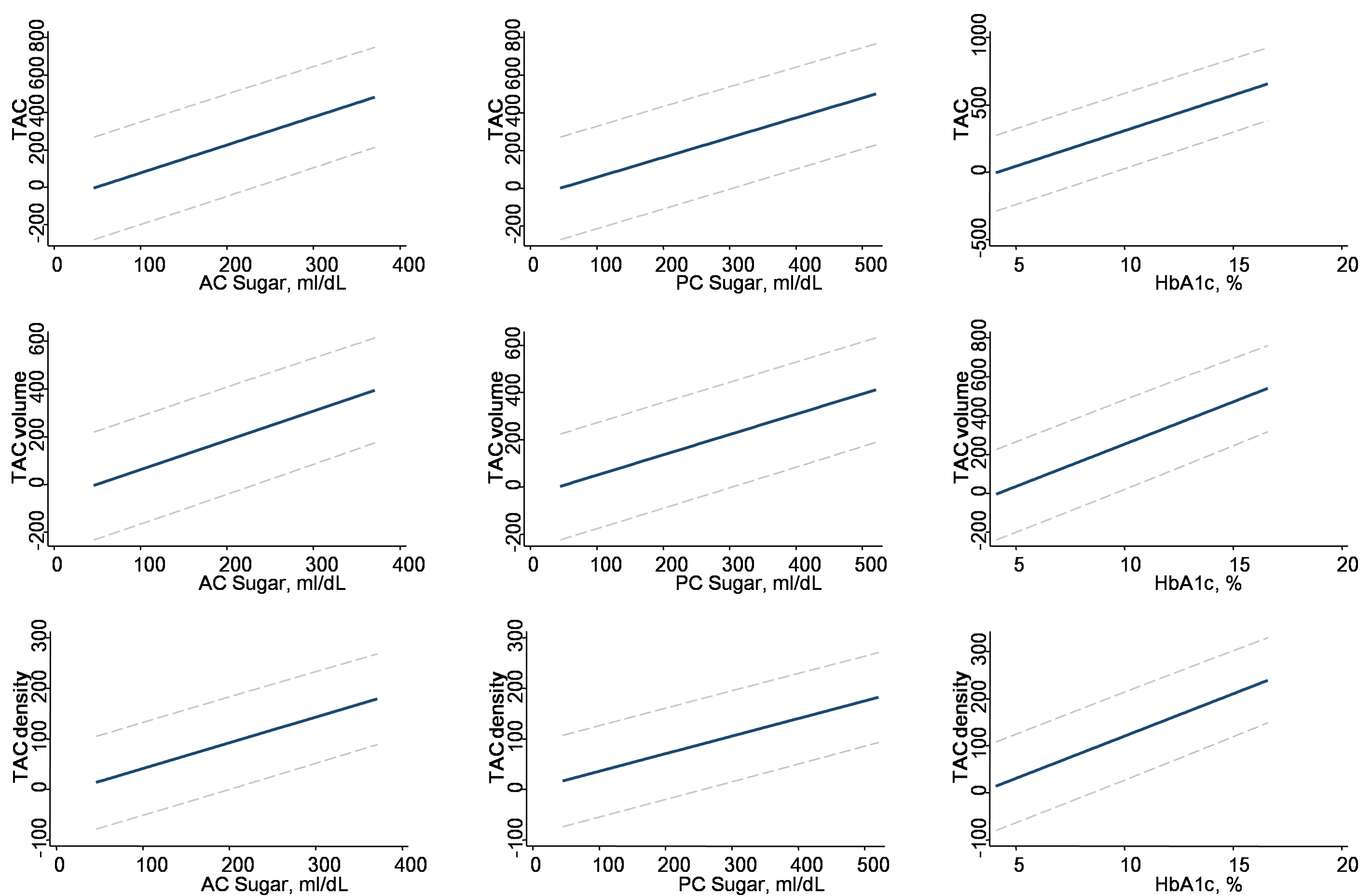

Supplement: S4 Fig — (TIF) [file pone.0207089.s004.tif]
