# Supplementary material for: Thoracic aortic calcification across the clinical dysglycemic continuum in a large Asian population free of cardiovascular symptoms
Source: PLoS One. 2019 Jan 4;14(1):e0207089. doi: 10.1371/journal.pone.0207089 (PMC6319708; doi:10.1371/journal.pone.0207089)
Supplement: S1 Table — (DOCX) [file pone.0207089.s005.docx]

**S1 Table.** Receiver operating characteristic curves and c-statistics of various dysglycemic indices on the presence of thoracic aortic calcification.

|  | **Presence of TAC** |
| --- | --- |
|  | **(TAC score, volume and Density>0)** |
| **c-statistics** |  |
| AC Sugar, mg/dL | 0.64 (0.61-0.67) |
| PC Sugar, mg/dL | 0.68 (0.65-0.71) |
| HbA1c, % | 0.68 (0.65-0.72) |
