# Supplementary material for: Thoracic aortic calcification across the clinical dysglycemic continuum in a large Asian population free of cardiovascular symptoms
Source: PLoS One. 2019 Jan 4;14(1):e0207089. doi: 10.1371/journal.pone.0207089 (PMC6319708; doi:10.1371/journal.pone.0207089)
Supplement: S2 Table — (DOCX) [file pone.0207089.s006.docx]

**S2 Table.** Comparison of differences between different levels of AC sugar and TAC related score.

|  | **AC Sugar: <100mg/dl)**  **(N=1783)** | **AC Sugar: 100~126mg/dl**  **(N=869)** | **AC Sugar: ≥126mg/dl**  **(N=140)** | **Diagnosed Diabetes Hx**  **(N=121)** | **P_trend_** |
| --- | --- | --- | --- | --- | --- |
| **TAC score** | 44.5 ± 346.6 | 84.9 ± 723.7^※^ | 246.4 ±1529.5.65^※¥^ | 384.2 ± 941.5^※¥^ | *<0.001* |
| **TAC volume** | 35.7 ± 275.3 | 69.6 ±569.2^※^ | 204.7 ±1230.9^※¥^ | 318.8 ± 779.3^※¥†^ | *<0.001* |
| **TAC density** | 28.6 ±84.0 | 52.2 ±108.1^※^ | 83.0 ±130.2^※¥^ | 131.3 ±143.3^※¥†^ | *<0.001* |

^※^Significant difference from non-diabetes, p<0.05;^¥^ Significant difference from pre-diabetes, p<0.05;^†^ Significant difference from undiagnosed diabetes mellitus , p<0.05.
