# Supplementary material for: Thoracic aortic calcification across the clinical dysglycemic continuum in a large Asian population free of cardiovascular symptoms
Source: PLoS One. 2019 Jan 4;14(1):e0207089. doi: 10.1371/journal.pone.0207089 (PMC6319708; doi:10.1371/journal.pone.0207089)
Supplement: S3 Table — (DOCX) [file pone.0207089.s007.docx]

**S3 Table.** Comparison of differences between different levels of PC sugar and TAC related score.

|  | **PC Sugar: <140mg/dl**  **(N=1807)** | **PC Sugar: 140~200mg/dl**  **(N=789)** | **PC Sugar: ≥200mg/dl**  **(N=131)** | **Diagnosed Diabetes Hx**  **(N=121)** | **P_trend_** |
| --- | --- | --- | --- | --- | --- |
| **TAC score** | 41.5 ± 270.9 | 107.0 ± 871.0^※^ | 284.3 ±1615.0^※¥^ | 384.2 ± 941.5^※¥^ | *<0.001* |
| **TAC volume** | 33.2 ± 206.2 | 85.9 ±678.1^※^ | 238.0 ±1319.5^※¥^ | 318.8 ± 779.3^※¥†^ | *<0.001* |
| **TAC density** | 32.2 ±87.2 | 54.6 ±111.9^※^ | 93.5 ±136.4^※¥^ | 131.3 ±143.3^※¥†^ | *<0.001* |

^※^Significant difference from non-diabetes, p<0.05;^¥^ Significant difference from pre-diabetes , p<0.05;^†^ Significant difference from undiagnosed diabetes mellitus , p<0.05.
