# Supplementary material for: Thoracic aortic calcification across the clinical dysglycemic continuum in a large Asian population free of cardiovascular symptoms
Source: PLoS One. 2019 Jan 4;14(1):e0207089. doi: 10.1371/journal.pone.0207089 (PMC6319708; doi:10.1371/journal.pone.0207089)
Supplement: S4 Table — (DOCX) [file pone.0207089.s008.docx]

**S4 Table.** Comparison of differences between different levels of HbA1C and TAC related score.

|  | **HbA1c: <5.7%**  **(N=1701)** | **HbA1c: 5.7-6.5%**  **(N=910)** | **HbA1c: ≥6.5%**  **(N=183)** | **Diagnosed Diabetes Hx**  **(N=121)** | **P_trend_** |
| --- | --- | --- | --- | --- | --- |
| **TAC score** | 36.6 ± 294.1 | 103.7 ± 908.4^※^ | 178.8 ± 741.8^※¥^ | 384.2 ± 941.5^※¥^ | *<0.001* |
| **TAC volume** | 29.0 ± 228.9 | 86.7 ±737.1^※^ | 141.8 ±505.5^※¥^ | 318.8 ± 779.3^※¥^ | *<0.001* |
| **TAC density** | 26.7 ±81.0 | 51.2 ±107.8^※^ | 89.9 ±132.5^※¥^ | 131.3 ±143.3^※¥^ | *<0.001* |

^※^Significant difference from non-diabetes, p<0.05;^¥^ Significant difference from pre-diabetes, p<0.05;^†^ Significant difference from undiagnosed diabetes mellitus , p<0.05.
